# Increased serum phenylalanine and tyrosine concentration related to inflammation in patients with primary angiitis of the central nervous system

**DOI:** 10.1016/j.bbrep.2026.102673

**Published:** 2026-06-16

**Authors:** Ning Feng, Jing Wang-Chang, Fen Wu, Han Su, Yaobo Zhao, Lina Sun, Shunfeng Zhao, Haoxiao Chang, Haowen Li

**Affiliations:** a China National Clinical Research Center for Neurological Diseases, Beijing Tiantan Hospital, Capital Medical University, Beijing, China; b Department of Clinical Laboratory, Liaocheng Third People's Hospital, Sub-Regional Center of China National Clinical Research Center for Neurological Diseases, Liaocheng, China; c First Clinical Medical College, Anhui Medical University, Hefei, China

**Keywords:** PACNS, Aromatic amino acids, Inflammation, Serum, Biomarker

## Abstract

**Background:**

Primary angiitis of the central nervous system (PACNS) is a rare disease characterized by severe central nervous system (CNS) vascular inflammation. Serum aromatic amino acids have been reported to associate with inflammation. However, the aromatic amino acids in PACNS are poorly understood.

**Methods:**

We obtained serum samples from 18 PACNS patients, 35 healthy control, and 17 other neurological diseases patients without inflammation. Serum concentrations of aromatic amino acids were assayed by a liquid chromatograph mass spectrometer (LC-MS). Inflammatory cytokine levels, including interleukin (IL)-1β, IL-6, IL-8, complement 3 (C3), C4, and tumor necrosis factor-α (TNF-α) were also collected.

**Results:**

Of aromatic amino acids, tyrosine, and phenylalanine but not tryptophan in serum were significantly elevated in PACNS patients. Notably, phenylalanine levels were positively correlated with C3, C4, and IL-8. Receiver operating characteristic (ROC) curve evidence tyrosine and phenylalanine are capable of discriminating between PACNS, HC, and OND.

**Conclusions:**

Our exploratory data suggest that elevated serum phenylalanine and tyrosine may be associated with PACNS-related inflammation. Whether these metabolites can serve as differential-diagnostic biomarkers requires validation in larger, multicentre cohorts.

## Highlights

• The serum level of Phe and Tyr were higher in the serum of PACNS patients.
• Serum Phe correlates positively with C3, C4, and IL-8 in PACNS patients.
• Serum Phe and Tyr show good diagnostic performance for discriminating PACNS.
• Aromatic amino acids may serve as exploratory biomarkers for PACNS.

## 1 Introduction

Primary angiitis of the central nervous system (PACNS) is an uncommon inflammatory disorder that impacts the brain and cervical spine, distinguished by pronounced central nervous system (CNS) vasculitis. The clinical manifestation is generally nonspecific, encompassing from headaches, focal neurological deficits, seizures, altered sensorium, cognitive dysfunction, and psychiatric symptoms to recurrent ischemic strokes, and a rare manifestation consists of myelopathy, optic neuritis, and ataxia [1,2]. In light of the rareness and nonspecific clinical presentation, the identification of PACNS mimics poses significant challenges in clinical practice, but essential to a severe CNS disease with considerable mortality [3,4]. The integration of clinical symptoms, imaging results, brain biopsy, laboratory and cerebral spinal fluid (CSF) analysis can diagnose PACNS, nonetheless, histological analyses of tissue acquired from a brain biopsy is recognized gold standard to ascertain definitive diagnosis [3,5]. The underlying pathobiology of PACNS remains poorly understood, although certain histopathological studies have suggested an immune-mediated inflammatory mechanism. This theory suggests that a variety of immune cells infiltrate cerebral vessels, leading to the development of distinct histopathological subtypes [1,4].

Aromatic amino acids (AAA), a subclass of α-amino acids characterized by an aromatic ring structure, comprise phenylalanine (Phe), tyrosine (Tyr), and tryptophan (Trp). Among these, Phe and Trp are classified as essential amino acids. Tyr is a semi-essential amino acid that is derived from Phe by phenylalanine hydroxylase [6]. Metabolic disorders of AAA have been reported to be associated with systemic inflammation and elevated pro-inflammatory cytokines [7,8]. Furthermore, their relationship with neuroinflammation and various other diseases has also been documented in previous studies [9,10]. However, the metabolic profile or concentration of AAA in patients with PACNS remains unelucidated. Based on the existing evidence, we hypothesize that AAA may be involved in the vascular inflammatory processes characteristic of PACNS.

This research seeks to examine the relationship between serum AAA concentrations and inflammatory cytokines profiles in PACNS patients, evaluating its potential diagnostic utility.

## 2 Materials and methods

### 2.1 Patients and sample collection

From 2017 to 2022, a total of 18 patients diagnosed with PACNS, 35 healthy controls (HC), and 17 patients with non-inflammatory neurological diseases (OND) were recruited from Beijing Tiantan Hospital. All PACNS cases were confirmed in the acute phase based on the diagnostic criteria established by Birnbaum and Hellmann [3,11]. The inclusion criteria for HC were lack of a history of inflammatory diseases, metabolic diseases, or any other underlying conditions. Diagnoses for patients were confirmed by biopsy or magnetic resonance imaging (MRI). Of the 18 PACNS patients, all showed an acute onset of symptoms. The OND group functioned as a negative control presented with various CNS disorders of non-inflammatory origin, excluding primary angiitis. The OND patients’ details are as following: three patients had cerebral venous sinus thrombosis, five had benign intracranial hypertension, two had peripheral neuropathy, two had meningeal cancer, one had intracranial hypotension headache, one had Paget bone disease, one had anxiety disorder, one had cranial nerve palsy, and one had idiopathic facial palsy.

We collected serum samples from patients during the acute stage, within two days of admission and prior to any treatment. The serum samples were proceeded to centrifuge them at 1000 g for 10 min, after which they were preserved at −80°C until analysis. A comprehensive data set was gathered by standard medical procedures for measuring interleukin (IL)-1β, IL-6, IL-8, complement 3 (C3), C4, and tumor necrosis factor-α (TNF-α) in our patient population. The corresponding clinical and demographic details, including the concentrations of AAA and a suite of cytokines and complement, are summarized in Table 1.

**Table 1:** Clinical characteristics、laboratory and imaging data of patients in the PACNS、HC and OND groups.

Subject details	PACNS(n = 18)	HC(n = 35)	OND(n = 17)
Gender [Male/Female (n)]^a^	14/4	19/16^ns^	10/7^ns^
Age (years, mean ± SD)^b^	33.00 ± 9.08	32.86 ± 9.66^ns^	36.65 ± 8.73^ns^
Serum Tyr [μmol/L, median (IQR)]^c^	199.50 (38.90,238.25)	40.70 (36.10,45.90)^#^	53.90 (37.95,90.80) ^ns^
Serum Phe (μmol/L, mean ± SD)^b^	79.84 ± 15.56	67.70 ± 12.29^#^	59.29 ± 10.72*
Serum Trp [μmol/L, median (IQR)]^c^	21.85 (18.08,37.30)	24.20 (17.30,32.60) ^ns^	22.70 (17.50,24.70) ^ns^
**Clinic Symptoms n (%)**			
Headache	8 (44.4%)		
Focal neurological deficits	11 (61.1%)		
Cognitive impairment	9 (50.0%)		
Epileptic seizure	12 (66.7%)		
Consciousness dysfunction	10 (55.6%)		
Psychiatric symptoms	4 (22.2%)		
Relapse n (%)	6 (33.3%)		
Prognosis [Good/Poor (n)]	10/8		
**Serum cytokines and complement**			
TNF-α [pg/mL, median (IQR)]	7.18 (6.44,26.75)		
IL-6 (pg/mL, mean ± SD)	2.20 ± 0.43		
IL-8 [pg/mL, median (IQR)]	33.50 (15.75,130.00)		
IL-1β (pg/mL, mean ± SD)	6.17 ± 2.90		
C3 (g/L, mean ± SD)	1.12 ± 0.26		
C4 (g/L, mean ± SD)	0.23 ± 0.06		
**Brain magnetic resonance imaging n (%)**			
Abnormal	18 (100%)		
Multiple hyperintense FLAIR lesions	18 (100%)		
Multiple bilateral infarcts	4 (22.2%)		
Intracranial hemorrhage	10 (55.6%)		
Abnormal lesion enhancement on MRI	18 (100%)		

^ns^ No significance. ^#^PACNS vs HC *p* < 0.05; *PACNS vs OND *p* < 0.05.

a Chi-squared test.

b One-Way ANOVA test.

c Kruskal-Wallis H test.

### 2.2 Chemicals and reagents

Reference substances for Phe, Tyr and Trp, together with their respective internal standards, were sourced from Sigma-Aldrich, (St. Louis, MO, USA). The ultra-pure water was deionised and filtered with a Milli-Q Plus system (Millipore Corporation, Bedford, MA, USA). All chemicals and reagents used in this study were obtained commercially at either analytical grade or LC-MS grade purity. Standard stock solutions were aliquoted in amber Eppendorf tubes and maintained at −80°C under light-protected conditions to ensure stability.

### 2.3 Sample preparation

Frozen serum specimens were thawed at a controlled temperature of 4°C. A volume of 20 μL of serum was supplemented with 80 μL of an internal standard mixture, which was composed of a commercial isotope-labeled amino acid standard (Sigma-Aldrich, catalog No. 767964-1 EA). The stock solution was diluted with the assay solvent to a working concentration of 5 μM (based on the lowest-concentration component). After thorough vortex mixing for 30 s, the sample was centrifuged at 20,000 rpm for 15 min at 4°C. The supernatant was isolated and aliquoted into a designated container. A specific volume of acidic water was subsequently then incorporated into the supernatant, ensuring thorough mixing, and the sample was allowed to equilibrate prior to analysis.

### 2.4 Measurement of serum aromatic amino acids

Referring to our previous research, the mass spectrum process and parameters in this study are as follows [12]. The liquid chromatography–tandem mass spectrometry (LC–MS/MS) system comprised a SCIEX QTRAP 6500+ triple quadrupole mass spectrometer (SCIEX, Foster City, CA, USA) interfaced with a Shimadzu Nexera X2 UHPLC system (Shimadzu Scientific Instruments, Columbia, MD, USA). The UHPLC system was equipped with a binary solvent delivery pump, thermostated autosampler, and column oven. Analyses were performed in positive ion mode using electrospray ionization (ESI) with the parameters: capillary voltage of 5.5 kV and desolvation temperature of 550°C. Chromatographic separation was achieved on an Intrada Amino Acid column (100 × 3 mm, 3 microm; Imtakt Corp, JAPAN) maintained at 40°C.The column temperature was established at 35°C, with mobile phase consisting of two solutions: Solution A, 100 mM Ammonium formate in water B, 0.1%FA in ACN. The mobile phase used to program the gradient elution was composed of A:100 mM Ammonium formate in water, B: 0.1%FA in ACN. The flow rate of 0.5 mL/min as follows: 0-0.5min, 80% B (v/v); 0.5-4.5min,70–80% B (v/v); 4.5–5min, 40-70% B (v/v); 5-10min, 0-40% B (v/v). At phase A 100% until 13min. Then, 13-13.2min return to the initial position 80% B (v/v). The system was restored to its original state within 0.5 min, followed by a re-equilibration period lasting 2 min. The sampler was thermostated at 4°C, and each injection consisted of 3 μL. The flow rate for elution was set at 500 μL/min. Quantitative analysis was performed in multiple reaction monitoring (MRM) mode. The optimized MS/MS parameters, including precursor/product ion transitions (Q1/Q3), declustering potential (DP), collision energy (CE), and cell exit potential (CXP) for each target analyte and its internal standard are summarized in Table 2. Data acquisition and processing were conducted using Analyst (version 1.7.3, SCIEX) and MultiQuant (version 3.2, SCIEX) software.

**Table 2:** Optimized MRM parameters for the quantification of aromatic amino acids.

Analyte	Q1 (*m*/*z*)	Q3 (*m*/*z*)	DP (V)	CE (eV)	CXP (V)
Phenylalanine	166.2	120.0	80	12	7
Tyrosine	182.2	136.0	90	13	7
Tryptophan	205.24	188.0	95	9	4

### 2.5 Statistical analysis

The statistical analysis was conducted using SPSS 26.0 and GraphPad Prism 9.4.1. Categorical variables were analyzed with chi-square tests. The Shapiro-Wilk method was used to test the normal distribution characteristics of the variables. For continuous variables that conformed to normal distribution, they were described by mean ± standard deviation (Mean ± SD), and comparisons between

Groups were analyzed using ANOVA analysis, followed by pairwise LSD comparisons; continuous variables that were not normally distributed were expressed as median and interquartile spacing [Median (IQR)], and were statistically analyzed using the Kruskal-Wallis H test with post-hoc multiple comparisons. When both variables were normally distributed, Pearson method was selected for correlation analysis. The Spearman correlation coefficient is a non-parametric method that does not assume a normal distribution of the data and is suitable for continuous data. Pearson's correlation was used to analyze correlations between Phe levels and serum C3 and C4, and the coefficient of Spearman's was utilized to assess the associations between Phe levels and serum TNF-α, IL-6, IL-8, and IL-1β and between Tyr levels and serum C3, C4, TNF-α, IL-6, IL-8, and IL-1β. The diagnostic performance of Tyr and Phe in predicting PACNS was assessed using receiver operating characteristic (ROC) analysis. Statistical significance was defined as *P* < 0.05.

## 3 Results

### 3.1 Patient characteristics

The clinical characteristics, laboratory parameters, and imaging data of patients in the PACNS (n = 18), OND (n = 17), and HC (n = 35) groups are presented in Table 1. Clinical manifestations were dominated by headache in 8 cases (44.4%), cognitive dysfunction in 9 cases (50.0%), focal neurological deficits in 11 cases (61.1%), seizures in 12 cases (66.7%), and psychiatric symptoms were predominant in 4 cases (22.2%). 12 patients underwent brain biopsy, and the pathological findings met the disease diagnostic criteria (Supplement Table 1). 6 patients without brain biopsy confirmation satisfied PACNS imaging criteria, revealing gadolinium-enhancing parenchymal lesions with vascular-related hyperintensity on FLAIR and T2-weighted MRI sequences. All patients with PACNS had vascular-related high-signal FLAIR and T2 lesions, and the MRI presentation met the imaging criteria for PACNS [3].

### 3.2 Comparison of serum AAA concentration between PACNS patients and control

**Fig. 1:**
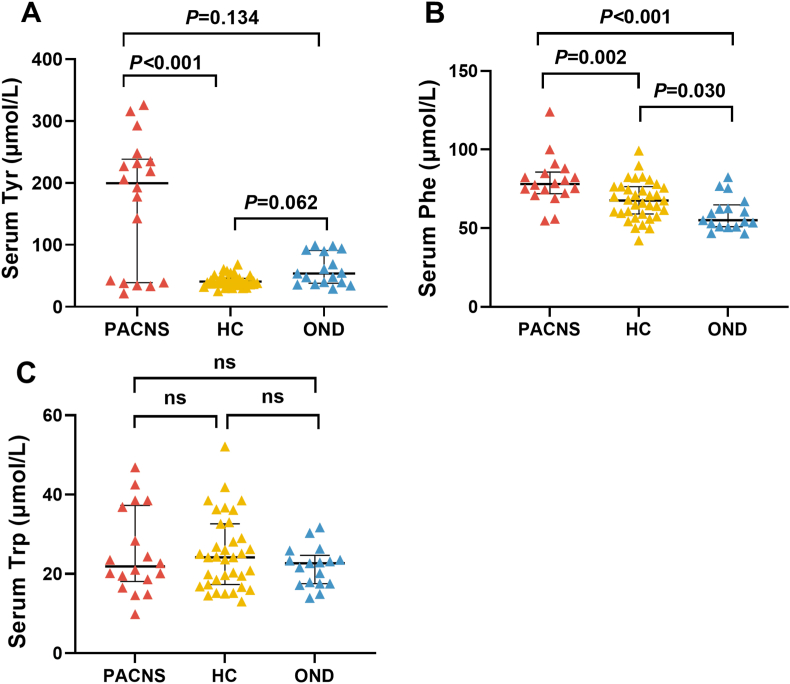
Comparison of Trp, Phe, and Tyr in serum between PACNS, HC and OND (A) The level of Tyr in PACNS group was significantly higher than that in healthy control group and OND group, (B) The level of Phe in the PACNS group were higher than those in the healthy control group, OND group, (C) The level of levels of Trp were not statistically different between PACNS group, healthy control, and OND group (*P* > 0.05). Normality was assessed by the Shapiro–Wilk test. One-way ANOVA followed by LSD post-hoc test was used for parametric variables (Phe and Trp), and Kruskal–Wallis H test followed by post-hoc multiple comparisons for non-parametric variables (Tyr). *P* < 0.05 was considered statistically significant. Tyr, tyrosine; Phe, phenylalanine; Trp, tryptophan.

The measurements of serum AAA in the PACNS (n = 18), OND (n = 17) and HC (n = 35) groups showed that serum Tyr level was significantly higher in the PACNS group compared to the HC groups (*P* < 0.001, Fig. 1A & Table 1). The Serum Phe levels were significantly higher in the PACNS group than in both the HC and OND groups (*P* = 0.002, *P* < 0.001; Fig. 1B). The Phe level was elevated in the OND group compared to the HC group (*P* = 0.030; Fig. 1B). Nevertheless, no statistically significant variation in serum Trp levels was seen among the three groups (Fig. 1C).

### 3.3 Correlations between serum AAA and inflammatory cytokines

**Fig. 2:**
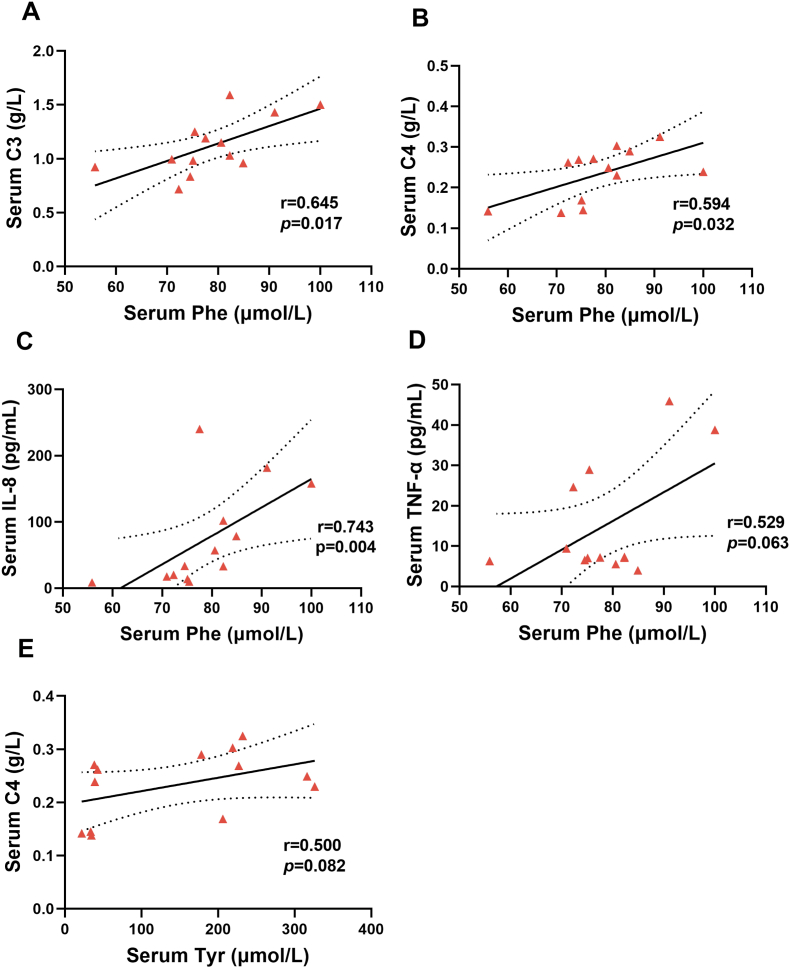
Correlation of serum Phe, Tyr and inflammatory factors (A-D) Phe levels in serum were positively correlated with C3, C4 and IL-8 (r = 0.645,*P* = 0.017; r = 0.594,*P* = 0.032; r = 0.743,*P* = 0.004) but not with TNF-a (r = 0.529,*P* = 0.063). (E) No correlation between serum Tyr and C4 (*P* > 0.05). Correlations were assessed using Pearson's correlation (Phe vs. C3/C4) or Spearman's rank correlation (Phe vs. IL-8/TNF-α; Tyr vs. inflammatory factors). Two-tailed tests were applied; *P* < 0.05 was considered statistically significant. Phe, phenylalanine; Tyr, tyrosine.

We next investigated the association between serum AAA levels and inflammatory cytokines. The concentration of Phe exhibited a positive correlation with serum C3 (r = 0.645, *P* = 0.017, Fig. 2A) and serum C4 (r = 0.594, *P* = 0.032, Fig. 2B). Additionally, the Phe concentration also demonstrated an excellent connection with serum IL-8 (r = 0.743, *P* = 0.004, Fig. 2C). A moderate association was identified between Phe concentration and serum TNF-α (r = 0.529, *P* = 0.063, Fig. 2D). We also examined the correlation between serum Phe concentration and serum IL-1β, but no significant association was observed (r = 0.052, *P* = 0.863, data not shown). In Fig. 1A, the serum Tyr levels was also higher in PACNS patients. We also investigated the relationship between serum Tyr levels and the aforementioned inflammatory cytokines in PACNS patients. However, serum Tyr levels showed no correlation with the serum C4 (Fig. 2E), C3, TNF-α, IL-6, IL-8, and IL-1β (data not shown).

### 3.4 ROC diagnostic value of serum Tyr and Phe for PACNS

**Fig. 3:**
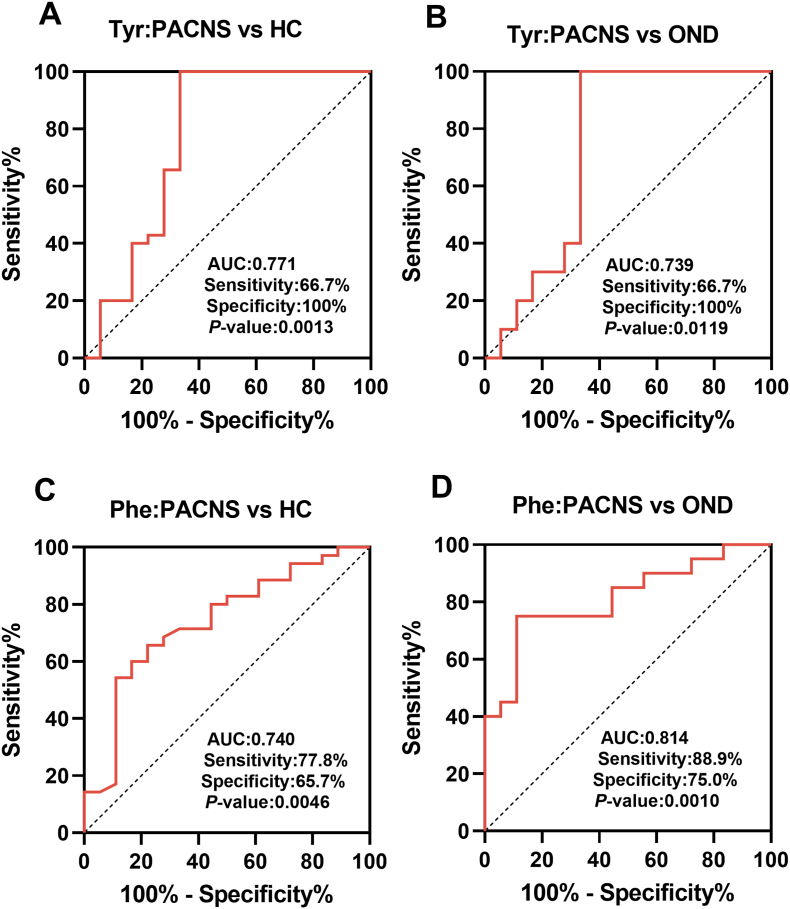
ROC curve analysis of predictive power of serum Tyr, Phe for PACNS (A) Tyr predicts the diagnostic value of PACNS group and HC group, (B) Tyr predicts the diagnostic value of PACNS group and OND group, (C) Phe predicts the diagnostic value of PACNS group and HC group, (D) Phe predicts the diagnostic value of PACNS group and OND group. The diagnostic performance of Tyr and Phe in predicting PACNS was assessed using receiver operating characteristic (ROC) analysis. Statistical significance was defined as *P* < 0.05. Optimal cutoff values were determined by the maximum Youden index (J = sensitivity + specificity − 1).

We further conducted a detailed examination to assess the diagnostic value of serum Tyr and Phe for identifying PACNS. The ROC curve analysis results revealed that the AUC for the diagnostic value of Tyr in PACNS patients compared to the HC group was 0.771. At the optimal critical value of 105.70, the sensitivity and specificity were 66.7% and 100%, respectively (*P* = 0.001, Fig. 3A). Similarly, the AUC for the diagnostic value of Tyr in PACNS patients compared to the patients in the OND group was 0.722. At the optimal threshold value of 120.90, the sensitivity and specificity were 66.7% and 100%, respectively (*P* = 0.025, Fig. 3B). For serum Phe, the AUC for the diagnostic value in PACNS patients compared to the HC group was 0.740. At the optimal threshold value of 71.75, the sensitivity and specificity were 77.8% and 65.7%, respectively (*P* = 0.005, Fig. 3C). In comparison, the AUC for the diagnostic value of Phe in PACNS patients compared to the OND group was 0.869. At the optimal critical value of 68.10, the sensitivity and specificity were 88.9% and 82.4%, respectively (*P* < 0.001, Fig. 3D).

## 4 Discussion

Our study indicated that the serum Tyr and Phe levels in PACNS group were obviously elevated compared among those in controls and non-inflammatory neurological disease groups. Moreover, according to our results, the serum Phe levels was significantly correlated to inflammatory cytokines (serum C3, C4, and IL-8). To our knowledge, it is first to report changes in serum Tyr and Phe levels in PACNS. And we studied the diagnostic significance of the serum Phe and Tyr concentration in PACNS. Those findings suggested that serum Phe and Tyr might be potential biomarker for clinical management in PACNS.

PACNS, a rare and severe form of vasculitis, exclusively affects the brain, spinal cord, and leptomeninges, as a result of vascular inflammation leading to cerebral ischemia, frequently accompanied by hemorrhage [13]. The main histopathological characteristics observed were granulomatous inflammation, infiltration by lymphocytes, and acute necrotizing vasculitis, which collectively demonstrate the inflammatory mechanisms underlying PACNS [14,15]. Our investigation revealed an increased trend of serum Tyr and Phe levels in PACNS patients when compared to control and OND groups. Further, we observed the correlation between the above two amino acids and inflammatory cytokines. These results supposed that AAA metabolism may be involved in the inflammatory response in PACNS.

As the fundamental building blocks of life, amino acids play critical roles not only in protein synthesis but also in multiple intracellular metabolic processes, such as ATP production, nucleotide biosynthesis, and maintenance of redox homeostasis, thereby supporting vital cellular and organismal functions [16]. Amino acids serve as vital nutrients for immune cells in organ development, tissue homeostasis maintenance, and execution of immune responses [17]. For example, succinate and itaconate are adept at accumulating within macrophages after LPS stimulation, subsequently influencing macrophage activation through either a positive or negative regulatory mechanism, respectively [18,19]. The IDO1 (Indoleamine 2,3-dioxygenase 1)-tryptophan-kynurenine-AhR (aryl hydrocarbon receptor) metabolic axis suppresses excessive pro-inflammatory activation in acute inflammatory settings; however, under chronic inflammation, this pathway promotes detrimental effects that facilitate the development of osteoporosis and vascular pathologies [20]. Currently more studies are expanding on the potential roles of Phe and Tyr metabolism in contributing to inflammation. Tyr, as a primary precursor for the dopamine synthesis, catabolic intermediate act as anti-inflammatory molecules by inhibiting inflammation through the suppression of inflammasome activation [21]. In sepsis, serum level of *meta*-tyr increase and peaks in the 2nd and 3rd days [22]. In a photo-aged skin model, Ishitsuka Y et al. found tyrosine act as an inflammation marker, increased during the process of inflammation induced by UV radiation [23]. Phe plays an important role in controlling the immune response to immune diseases. Rober Zangerle et al. indicated that higher Phe to Tyr ratio in blood correlated to immune activation and inflammation in HIV-1 infection patients [24]. Moreover, Phe dictates the effector functions of immune cells. Phe regulates T cell proliferation and activation [25] and diminishes M1 macrophage inflammation by inhibition of the production of interleukin (IL)-1β and TNF-α [26]. The increases in Phe concentrations at the expense of Trp also associate with the chronic low-grade inflammation in aging persons [27]. These results supposed that Phe and Tyr metabolism are involved in inflammation, which supported our results.

Our study also observed that both serum levels of Phe and Tyr are related to the inflammatory cytokines. The serum Phe levels are both positive related to the serum complement (C3 and C4) and following serum cytokines:IL-8. However, the serum Tyr levels were not related with the above inflammatory cytokines. The complement system plays an essential role in the innate immune system and can exacerbate immune inflammation​ [28]. Pinni Yang et al. observed that serum complement C3 levels have been associated with ischemic stroke [29]. Besides, the complement system activation is a causal factor for the human autoimmune disease, especially in the renal vasculitis [30]. Thus, we selected serum complement C3 and C4, to see the relationship between the serum Phe levels and the complement system. Phe is involved in the C3 fragments binding on neutrophils and monocytes to induce a neutrophil activation, which may explain only serum Phe levels positively correlated with serum complement C3 and C4, not serum Tyr levels [31]. Besides, IL-8 also gets involved in the neutrophil activation, by changing the expression of neutrophil surface-receptor [32]. This may further explain serum Phe levels also positive correlated with serum IL-8 levels in our study. The amino acids metabolism and inflammatory factors has a complex network relationship. The indoleamine 2,3 dioxygenase (IDO) subset of the kynurenine (KYN) (IDO/KYN), is a metabolic inflammatory pathway involved in production of nicotinamide adenine dinucleotide (NAD+) [33,34]. It has been shown that IDO/KYN actively participates in inflammatory processes and can increase the secretion of cytokines that provoke inflammatory diseases. Amino acid metabolites in HIV-infected people with metabolic problems are associated with inflammation cytokines, including TNF-α, C-reactive protein, and IL-1β [35]. In cancer cachexia patients, inflammatory cytokines such as IL-6 and TNF-α contribute to the effects of inflammation on protein metabolism. These results supposed that amino acids metabolism get involved in inflammation [36].

The quantitative analysis of serum phenylalanine and tyrosine in this study was performed using LC-MS/MS. This technique was selected for its superior sensitivity, specificity, and ability to simultaneously and accurately quantify multiple target metabolites without the requirement for derivatization, which is particularly advantageous for the precise measurement of amino acids in complex biological samples like serum [37,38]. The reliability of our metabolite data, and consequently the subsequent biomarker analysis, is underpinned by this robust analytical approach. Compared with PCR, ELISA, results from LC-MS/MS are more accurate and stable, so mass spectrometry method is adopted in this study.

We also analysis the ROC curve of serum levels Phe and Tyr to detect the prediction power for PACNS. Both serum levels of Phe and Tyr has the potential predictive value as diagnostic biomarkers.

In our study, we found that both serum Tyr and Phe levels had statistical differences when compared healthy control group. The mean ± standard deviation (SD) for data of phe in healthy controls was 67.70 ± 12.29 μmol/L, while in another non-inflammatory neurological group was 59.29 ± 10.72 μmol/L, showing a slightly down trend. We supposed that the cause of statistical difference may be related to some non-specific metabolic disorders of the patients.

Several limitations cannot be ignored in our study. First, our study is a single-center investigation, which may be subject to biases related to the limited sample size and varying follow-up durations. It is important to note that recruiting a large cohort remains challenging for a rare neurological disorder such as PACNS. Secondly, serum cytokine and complement component data for the PACNS group were obtained from routine clinical practice, whereas such data are not available for the HC and OND groups. This limitation precludes us from determining whether the observed correlation between metabolites and serum cytokine/complement component levels is specific to PACNS or reflects a more general interaction between amino acids and immune activity. In future studies, further investigation is needed to elucidate the pathways through which phenylalanine participates in the inflammatory response of PACNS, as well as to advance our understanding of the disease's pathogenesis. In addition, the reported correlations between Phe and inflammatory markers are modest and purely associative, without evidence for causality.

Overall, our study provides evidence from the viewpoint of AAA metabolism, highlighting the association between Phe and Tyr inflammation in PACNS. The findings from the study indicate that AAA are involved in the development of the disease and the associated inflammatory process, thereby providing a novel approach to potential therapeutic targets and diagnostic biomarkers.

## 5 Conclusions

Serum AAA are markedly elevated in PACNS patients. This finding suggests that serum AAA may be linked to PACNS pathogenesis and show a positive association with pro-inflammatory factors. Therefore, serum AAA may serve as potential exploratory biomarkers for monitoring disease activity and supporting diagnosis.

## Ethics approval and consent to participate

Experiments were carried out following the ethical principles established in the Declaration of Helsinki. Ethical approval for this study was granted by the Ethics Committee of Beijing Tiantan Hospital, Capital Medical University (Approval No. KY2022-15-01). Every participant signed an informed-consent form prior to enrolment.

## Consent for publication

All authors unanimously approved the final version of the manuscript.

## Funding

This study was supported in part by the 10.13039/501100001809National Science Foundation of China (82301453).

## CRediT authorship contribution statement

**Ning Feng:** Data curation, Validation, Visualization, Writing – original draft. **Jing Wang-Chang:** Data curation, Formal analysis, Investigation, Software, Visualization. **Fen Wu:** Investigation, Software, Validation. **Han Su:** Formal analysis, Software. **Yaobo Zhao:** Methodology. **Lina Sun:** Formal analysis, Software. **Shunfeng Zhao:** Conceptualization, Supervision. **Haoxiao Chang:** Conceptualization, Investigation, Project administration, Validation, Visualization, Writing – original draft, Writing – review & editing. **Haowen Li:** Funding acquisition, Resources, Software, Supervision, Writing – review & editing.

## Declaration of competing interest

The authors state no potential conflicts of interest with regard to the commercial or financial aspects of this research.

## Data Availability

Data will be made available on request.
