# Supplementary material for: Increased serum phenylalanine and tyrosine concentration related to inflammation in patients with primary angiitis of the central nervous system
Source: Biochem Biophys Rep. 2026 Jun 16;47:102673. doi: 10.1016/j.bbrep.2026.102673 (PMC13284509; doi:10.1016/j.bbrep.2026.102673)
Supplement: Multimedia component 1 [file mmc1.pdf]

Supplementary Table 1. Biopsy pathological features of 12 PACNS patients.

No. patient	Histological features
1	Perivascular infiltration of inflammatory cells, including T, B cells and macrophages
2	Microvascular hyperplasia and infiltration of inflammatory cells
3	Perivascular infiltration of inflammatory cells
4	Perivascular infiltration of inflammatory cells
5	Perivascular infiltration of inflammatory cells, including T, B cells and macrophages
6	Lymphocyte infiltration scattered around blood vessels
7	Space occupying vasculitis-like lesion in the right parietal lobe
8	Middle frontal gyrus inflammatory lesions
9	Perivascular infiltration of inflammatory cells
10	Perivascular infiltration of inflammatory cells
11	Transmural vasculitis
12	Perivascular infiltration of inflammatory cells
